# Expression of prolactin receptors in normal canine mammary tissue, canine mammary adenomas and mammary adenocarcinomas

**DOI:** 10.1186/1746-6148-8-72

**Published:** 2012-05-30

**Authors:** Erika Michel, Stefanie K Feldmann, Mariusz P Kowalewski, Carla Rohrer Bley, Alois Boos, Franco Guscetti, Iris M Reichler

**Affiliations:** 1 Clinic for Reproductive Medicine, Unit of Small Animal Reproduction, Vetsuisse Faculty, University of Zurich, Winterthurerstrasse 260, 8057, Zurich, Switzerland; 2 Institute of Veterinary Anatomy, Vetsuisse Faculty, University of Zurich, Winterthurerstrasse 260, 8057, Zurich, Switzerland; 3 Division of Radiation Oncology, Vetsuisse Faculty, University of Zurich, Winterthurerstrasse 260, 8057, Zurich, Switzerland; 4 Institute of Veterinary Pathology, Vetsuisse Faculty, University of Zurich, Winterthurerstrasse 260, 8057, Zurich, Switzerland

**Keywords:** Dog, Canine, Mammary tumor, Prolactin, Prolactin receptor

## Abstract

**Background:**

Mammary tumors represent the most common neoplastic disease in female dogs. Recently, the promoting role of prolactin (PRL) in the development of human breast carcinoma has been shown. Possible proliferative, anti-apoptotic, migratory and angiogenic effects of PRL on human mammary cancer cells in vitro and in vivo were suggested. The effects of PRL are mediated by its receptor, and alterations in receptor expression are likely to play a role in tumor development. Currently, not much data is available about prolactin receptor (PRLR) expression in canine mammary tumors. To set the basis for investigations on the role of PRL in mammary tumorigenesis in this species, prolactin receptor expression was evaluated by semi-quantitative real time PCR and immunohistochemistry on 10 formalin-fixed, paraffin-embedded samples each of canine non-neoplastic mammary tissue, mammary adenomas and adenocarcinomas.

**Results:**

The highest PRLR expression levels were found in normal mammary tissue, while adenomas, and to an even higher degree adenocarcinomas, showed a significant decrease in prolactin receptor expression. Compared to normal tissue, PRLR mRNA was reduced 2.4 fold (*p* = 0.0261) in adenomas and 4.8 fold (*p* = 0.008) in adenocarcinomas. PRLR mRNA expression was significantly lower in malignant than in benign lesions (*p* = 0.0165). Immunohistochemistry demonstrated PRLR expression in all three tissue types with signals mostly limited to epithelial cells.

**Conclusions:**

Malignant transformation of mammary tissue was associated with a decline in prolactin receptor expression. Further studies are warranted to address the functional significance of this finding.

## Background

Prolactin (PRL) is a polypeptide hormone synthesized in and secreted by anterior pituitary lactotrophic cells. In addition, other tissues including the central nervous system, immune system, uterus and even the mammary gland are known to produce PRL in humans [1]. In most mammalian species, PRL is involved in proliferation and differentiation of normal breast epithelium and in stimulating post partum lactation. The biological actions of PRL are not limited to its essential role in reproduction, but also involve regulation of the immune system, osmotic balance, angiogenesis and behaviour [1]. In the female dog, as well as in some rodent species, PRL is an essential luteotrophic hormone in the second half of pregnancy and is also involved in the display of maternal behavior [2-4]. PRL plays a promoting role in the development of human breast carcinoma, in addition to a variety of benign breast lesions [5-8]. It has been shown to exert – in an estrogen independent manner – proliferative, anti-apoptotic, migratory and angiogenic effects on human mammary cancer cells and tissues in vitro and in vivo [9]. The effects of PRL are mediated by its receptor [1]. PRL receptor (PRLR) expression is found in 70–100% of all mammary tumors and in 93-100% of normal breast tissue biopsies by means of RT-PCR and in-situ hybridization [10-14]. Some studies reported PRLR expression to be higher in malignant breast lesions compared to normal tissue [12,15], but others did not confirm this finding [11,16,17]. In breast cancer cell culture models, PRLR expression was found to be increased, decreased or unaltered compared to normal breast epithelium, depending on the cell line used [10,18]. A few authors described PRLR expression at different subcellular localizations in normal, benign and malignant breast epithelial cells: In healthy cells expression seemed to be limited to the luminal borders, whereas malignant epithelial cells mainly showed cytoplasmatic expression. In benign lesions, variable degrees of luminal and cytoplasmatic expression were seen [13,15].

Spontaneously occurring canine mammary tumors show similar epidemiological, pathological and biochemical characteristics as human breast cancer, although the relative frequency of different histological subtypes is very different [19-24]. The incidence in female dogs is threefold higher than in women, and mammary neoplasms are the most common tumor type of the intact female dog and account for approximately 50% of all neoplasms in bitches [25]. As many as 41-53% of the tumors are malignant, and in these cases the 2-year survival is only about 25-40% [25,26]. Incidence increases with age: the median age of tumor manifestation is 10 to 11 years [27,28]. A variety of different factors play a role in canine mammary tumorigenesis, but hormones are, beside age, the most important [29]. The influence of the sexual steroids estrogen and progesterone on the pathogenesis of canine mammary tumors has been studied extensively [23,28-30]. While steroid hormones are considered to be involved in early carcinogenesis, they seem to lose their stimulatory effect during progression of disease [31]. The role of PRL in tumorigenesis of canine breast cancer is not known to date. Sporadic publications proposed a tumor-promoting role [32,33], and presence of PRLR was documented in canine mammary tumor cells in vivo and in vitro [34,35]. Also were serum and tissue homogenate levels of PRL found to be elevated in dogs with benign and malignant mammary tumors [36]. However, data is incomplete, inconsistent and partially methodologically outmoded.

The aim of this study was to expand our current knowledge about the influence of PRL on canine mammary tumorigenesis by evaluating PRLR expression in non-neoplastic canine mammary tissue and in benign and malignant mammary tumors by means of semi-quantitative real time (TaqMan) PCR. Immunohistochemistry was used to identify the main cells types expressing PRLR in these tissues.

## Methods

### Tissues

Archival normal and neoplastic mammary tissue that had been fixed in buffered 10% formalin for 24 to 72 h, sent to the Institute of Veterinary Pathology, Vetsuisse Faculty, University of Zurich by the Unit of Small Animal Reproduction, Vetsuisse Faculty, University of Zurich, and embedded in paraffin by routine procedures was used. Selected samples originated from 25 intact female dogs of different breeds, which had undergone surgery, consisting either of a regional or a radical mastectomy, due to mammary gland tumor. At the time of surgery, the dogs were aged between 5 and 13 years. Any experimental research was approved by the Cantonal Veterinary Office of Zurich.

For the present study, ten samples each of normal mammary tissue, mammary adenomas and mammary adenocarcinomas, respectively were selected from the paraffin blocks of the 25 dogs. Selection was based on state of preservation and histopathological diagnosis. Two punch biopsies with a diameter of 2.0 mm were collected manually from each paraffin block at sites selected using HE stained sections (Tissue MicroArray Builder, cat no. 20010.2, punch-extractor “pen”, Histopathology Ltd, H-7608 Pécs). Special attention was paid to removing only the desired tissue type. These two punch biopsies were embedded side by side in a new paraffin wax block. From these blocks, the following consecutive sections were cut using a rotary microtome (RM 2165, Leica, Germany): 1 × 3 μm section for HE staining, 20 × 10 μm sections for RNA extraction and real time PCR (see below), 3 × 3 μm sections for immunohistochemistry (see below), 1 × 3 μm section for HE staining. The HE stained sections served to make sure that only the desired tissue type had been harvested; this staining was done using routine methods.

### Semi-quantitative real time (TaqMan) PCR

Excessive paraffin was manually removed using needles from the sections floating on a water bath before transferring all sectioned tissue of each block into a 1.5 ml PCR tube (Eppendorf, Germany). RNA was extracted using the RNeasy FFPE Kit (Qiagen, Switzerland) according to the manufacturer’s instructions. RNA concentration was measured UV-photometrically (SmartSpec, BioRad, Switzerland). RNA was stored at −20°C until analysis.

RNA was treated with recombinant, RNase-free DNase I to eliminate genomic DNA contaminations (Roche Molecular Biochemicals, Mannheim, Germany), according to the manufacturer’s instructions. Reverse transcription reagents were supplied from Applied Biosystems, Foster City, CA, USA. Reactions were performed using random hexamers as primers for the cDNA synthesis. 100 ng of DNase-treated total RNA was used for each sample. Semi-quantitative Real Time (TaqMan) PCR was carried out in an automated fluorometer (ABI PRISM® FAST 7500 Sequence Detection System, Applied Biosystems, Foster City, CA, USA) using 96-well optical plates (Eurogentec, Belgium) according to the previously described protocol [37,38]. Samples were run in duplicates with Fast Start Universal Probe Master (ROX) from Roche Diagnostic. In order to make sure that the reagents used for RT-PCR reactions were not contaminated and to confirm the accuracy of the DNase treatment, we used autoclaved water instead of RNA and the so called RT minus control as negative controls. The primers as well as the 6-carboxyfluorescein (6-FAM) and 6-carboxytetramethylrhodamine (TAMRA) labeled probes were ordered from Eurogentec, B-4102 Serain, Belgium. Selection was done with the aid of the Primer Express Software (Version 2.0, Applied Biosystems, USA).

Relative quantification was done by normalizing the signals of the target gene with those of a housekeeping gene (GAPDH) and using the comparative CT method (ΔΔ CT method) according to the instructions of the manufacturer of the ABI PRISM® 7500 Sequence Detector and as previously described by Kowalewski et al. [38]. Data were considered valid if the relative amount of the reference gene for a sample was constant (i.e. was similar in both duplicates) and the average amount was used as a normalization factor in the ΔΔ CT method. Specificity of selected PCR products was confirmed by sequencing (Microsynth, Switzerland).

Sequences for primers and TaqMan probes were as follows:

*GAPDH* (forward): 5′- GCT GCC AAA TAT GAC GAC ATC A-3′

*GAPDH* (reverse): 5′- GTA GCC CAG GAT GCC TTT GAG-3′

*GAPDH* (TaqMan Probe): 5′- TCC CTC CGA TGC CTG CTT CAC TAC CTT-3′

*PRLR* (forward): 5′- GGA TCT TTG TGG CCG TTC TTT-3′

*PRLR* (reverse): 5′- AAG GAT GCA GGT CAC CAT GCT AT- 3′

*PRLR* (TaqMan Probe): 5′- ATT ATG GTC GTA GCA GTG GCT TTG AAA GGC-3′

For statistical data evaluation the software GraphPad 3.06 (GraphPad Software Inc, San Diego, CA, USA) was used. Due to the uneven distribution of the Real Time PCR results, the data obtained are presented as geometric means with deviation factor (Xg x DF^±^1). An unpaired *t*-test was performed. *P*-values < 0.05 were considered to be significant.

### Immunohistochemistry

A standard immunoperoxidase immunohistochemical procedure was applied as previously described [37-39]. Briefly, from each paraffin block, 3 sections of 3 μm were cut and mounted on SuperFrost Plus microscope slides (Menzel-Gläser, Braunschweig, Germany). The antibody used was goat affinity purified polyclonal anti human PRLR IgG (R&D Systems, USA) diluted 1:50. This antibody was chosen after preliminary tests out of a panel of four antibodies (including the polyclonal H-300 (sc-20992, Sta. Cruz Biotechnology) and the monoclonal antibodies U5 (SM5033P, Acris, Germany) and B6.2 (AM00543PU-N, Acris, Germany) due to its good signal-to-background ratio in a tissue array containing canine non-neoplastic mammary tissues, mammary adenomas and adenocarcinomas, as well as pituitary tissue. The other three antibodies were discarded because of less consistent labeling (U5), complete absence of labeling (B6.2), or predominance of unspecific labeling (H-300). Sections incubated without antibody or with the corresponding isotype control (irrelevant goat IgG, I-5000, Vector Laboratories, USA) were used as negative controls.

Evaluation of sections was performed microscopically at 200x and 400x magnification. Negative controls were used to differentiate between non-specific or false-positive staining reactions. Evaluation of PRLR expression was performed descriptively.

## Results

### Semi-quantitative real time (TaqMan) PCR

**Figure 1:**
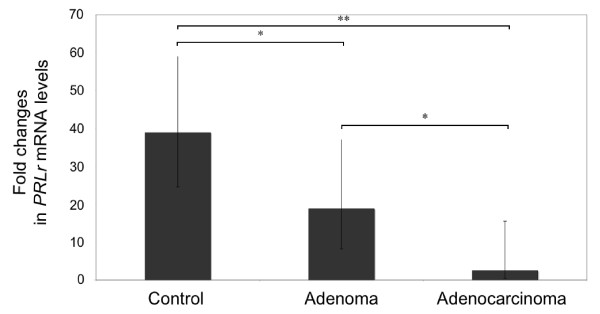
** Expression of PRLR in normal canine mammary tissue, benign (mammary adenomas) and malignant (mammary adenocarcinomas) lesions as determined by Real Time (TaqMan) RT-PCR (normalized to GAPDH expression): mean (Xg x DF ± 1); bars with one asterisk differ at (*P* < 0.05); bars with two asterisks differ at (*P* < 0.01).**

By means of semi-quantitative Real Time PCR, the highest PRLR expression levels were observed in non-neoplastic mammary tissues. In adenomas, expression was significantly reduced compared to non-neoplastic tissues (*p* = 0.026). Adenocarcinomas as well had significantly reduced PRLR expression levels compared to non-neoplastic tissues (*p* = 0.008). PRLR mRNA expression was significantly lower in adenocarcinomas compared to adenomas (*p* = 0.017). Overall, PRLR expression was 2.4 fold reduced in adenomas and 4.8 fold reduced in adenocarcinomas compared to normal tissues (Figure 1).

### Immunohistochemistry

**Figure 2:**
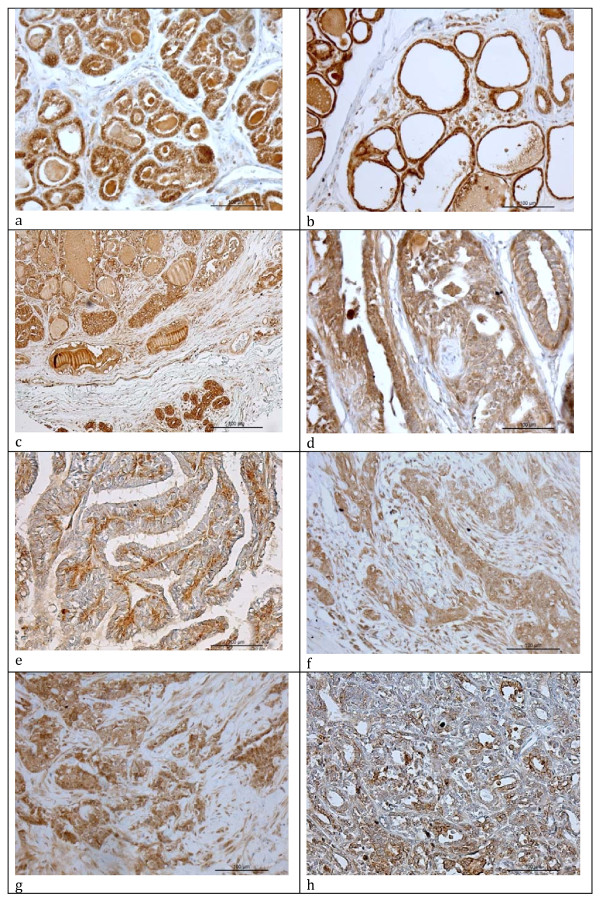
** Immunohistochemical labeling of PRLR in canine mammary tissues. a**) Non-neoplastic mammary tissue. Signal can be detected in epithelial cell cytoplasm and in alveolar secrete. Connective tissue cells are mostly negative. **b**) Non-neoplastic mammary tissue. Epithelial cells are flattened and exhibit a strong signal. **c**) Mammary adenoma. The adjacent non-neoplastic tissue shows more intense positive staining than the neoplastic one. This tissue is from an original block used for the study. **d**) Mammary adenoma. Signal is present in epithelial cells. **e**) Mammary adenoma. Staining is mainly localized to the basal epithelial cell layer. **f**) Mammary adenocarcinoma. Signal is present in epithelial cells and rarely in some fibrous tissue cells. **g**) Mammary adenocarcinoma. An intense signal is present in epithelial cells. **h**) Mammary adenocarcinoma. Only a weak signal signal is present in epithelial cells. Immunoperoxidase staining, anti-human PRLR antibody.

Expression of PRLR protein was demonstrated in canine mammary tumors and in non-neoplastic canine mammary tissues using a polyclonal goat anti human PRLR antibody (R&D Systems, USA). Specific signal was detected in non-neoplastic tissues (Figure 2a, b), adenomas (Figure 2c-e) and adenocarcinomas (Figure 2f-h).

#### Non-neoplastic mammary tissues

PRLR positivity was observed in all samples examined in form of dark brown staining in different compartments in mammary epithelial cells comprising the cytoplasm and cell membranes, especially along the luminal and lateral borders (Figure 2a). In two cases, labeling of the nuclei was detected. Overall, the signal was very intense in epithelial cells, while it was mostly absent from myoepithelial cells and connective tissue cells. Blood vessels (endothelial cells and smooth muscle cells) and inflammatory cells were partly labeled and partly negative. Secrete in the alveoli was strongly positive in all sections.

#### Benign lesions: mammary adenomas

All samples showed PRLR positivity. Most samples showed a slightly less intense labeling than seen in non-neoplastic mammary gland tissue (Figure 2c). In four samples, intensity of staining was comparable with normal tissue. Signals were mainly detected in epithelial cells and, to a lesser extent, in some myoepithelial cells. Often, signals were detected at the luminal cell borders in addition to cytoplasmic labeling (Figure 2c, d). In one case, labeling was most intense in the basal epithelial cell cytoplasma, while the luminal epithelial compartments gave weaker signals (Figure 2e). Connective tissue cells showed slightly more often signals than in non-neoplastic mammary tissues. Blood vessels were partly labeled and partly negative, as in non-neoplastic mammary tissues. Some reactivity was also detected in inflammatory cells.

#### Malignant lesions: mammary adenocarcinomas

All examined cases showed PRLR positivity (Figure 2f-h). Intensity of labeling was in most cases comparable with benign lesions. Two cases were stained more intensely than normal tissue. Labeling was most intense in epithelial cells, while only a few signals were detected in the connective tissue.

## Discussion

Our study evaluates for the first time the canine PRLR expression in non-neoplastic, benign and malignant canine mammary tissue both at the protein (immunohistochemistry) and the mRNA level (semi-quantitative real time PCR). Expression was detected in all tissue types, and expression levels were highest in normal mammary epithelial tissue, as demonstrated quantitatively by real time PCR. Moreover, real time PCR showed a significant reduction in PRLR expression both in benign neoplastic lesions and even more marked in malignant lesions.

To the authors’ knowledge there are only two other studies analyzing PRLR expression in canine mammary tumor cells [34,35]. Rutteman et al. also found reduced PRLR expression in malignant mammary tumors compared to benign ones [34]. In this study, all cases of normal tissue and benign lesions were receptor positive, but positivity was found only in a few malignant lesions [34]. The radioimmunoassay technique used involved binding of radiolabeled PRL to tissues in order to detect the presence and quantify the relative expression level of PRLR. However, in the meantime, modern PCR-based approaches were shown to be more specific and more sensitive in human breast cancer studies [40]. Van Garderen et al. used RT-PCR to demonstrate expression of PRLR in the canine mammary tumor cell line CMT-U335 [35]. But the expression rate was not quantitatively compared to normal canine mammary epithelial cells. In most studies evaluating human breast cancer, PRLR expression level in malignant tumors was equal or decreased compared to benign lesions and normal tissue [11,13,16,17]. However, a few studies demonstrated a higher PRLR expression in malignant lesions [12,15]. The heterogeneous subcellular distribution of the PRLR (variously including cytoplasm, nucleus, sometimes luminal borders of the epithelial cells), labeling of myoepithelial cells and weak labeling of individual connective tissue cells found in our study is in agreement with most human medicine reports [11,12,16,17,41]. In contrast to the findings of Gill et al. and Ferreira et al., we were not able to demonstrate a variation of PRLR expression at different subcellular locations between non-neoplastic cells and benign and malignant mammary tumor cells [13,15]. Gill et al. found that PRLR expression was mainly limited to luminal cell borders in normal female mammary tissue, while it was both luminal and cytoplasmatic in benign lesions and mostly cytoplasmatic in malignant lesions. Ferreira et al. reported similar results in pathological tissues from male individuals. In gynecomastia, PRLR expression was mainly seen along luminal cell borders, while in mammary carcinomas from males signals were mostly cytoplasmatic [13,15]. Similar to others, we observed staining of endothelial cells and smooth muscle cells of some intratumoral blood vessels, which might be related to the angiogenic effect of prolactin [11,17].

Queiroga et al. investigated PRL tissue levels in tissue homogenates of normal canine mammary tissue, benign and malignant mammary tumors. They found PRL tissue levels to be significantly associated with the malignancy of tumors, with malignant tumors expressing the highest levels [36]. However, it remains to be demonstrated whether PRL tissue levels correlate with PRLR status. Neither PRLR nor possible local PRL expression was analyzed in this study [36].

A plausible explanation for the fact that PRLR expression was highest in canine normal mammary tissue and lowest in malignant tumors is that PRLR expression may be a differentiation marker of mammary epithelial cells and that loss of this marker may be a characteristic of dedifferentiation. While PRL acts as a pro-oncogene in early neoplastic transformation and is certainly involved in cellular neoplastic progression and resistance to breast cancer treatment, it is also well characterized as a terminal differentiation factor for mammary epithelial cells and as essential regulator of epithelial plasticity and invasion and metastasis suppressor hormone [8,9,42-47]. Similarly, steroid hormone receptors are considered cell differentiation markers as well, and their expression decreases with increasing malignancy of canine mammary tumors, and is absent in metastases [20]. In this context, the findings of Queiroga et al. are surprising, because not only PRL levels, but also steroid hormone levels were higher in tissue homogenates of malignant tumors compared to benign lesions and non-neoplastic tissue [36]. It should be noted, however, that an increase of the local concentration of hormones does not necessarily need to be associated with increased expression of their receptors in tumor tissues. Even a very low expression level of the receptor is sufficient to mediate PRL responsiveness in human breast cancer cell lines [40]. Furthermore, alterations of intracellular PRL signaling could be crucially involved in carcinogenesis, as PRL signaling pathways have an essential role in maintaining physiological cell differentiation and in the regulation of cell cycle and apoptosis [14,47-49].

Small sample size was the major limitation of this study. To exclude external variables as well as possible, only tissue samples from intact female dogs were included. Unfortunately, it was not possible to retrospectively identify the exact stage of estrous cycle in the bitches due to the lack of a gynecological examination, vaginal cytology and hormone assays [50]. It seems however highly unlikely that the mammary tumors used in this study were removed during estrus, i.e. under estrogen influence, because all patients were clinically diagnosed as not in heat. Therefore, it cannot be excluded with certainty that some of the tumors were removed during metestrus, i.e. under progesterone influence. In the female pig, only estrogen, but not progesterone alone or in combination with estrogen has an effect on PRLR expression [51]. Since the stage of estrous cycle was not determined in any of the animal or human studies (analyzing pre-menopausal women) known to the authors, we consider the data presented in this work to be comparable to previous investigations. Another possible limitation of this study is the fact that we used non-affected mammary tissue from dogs with mammary tumors as normal samples. Even if this approach is consistent with Rutteman et al. 1986 [34] and many human studies, which also compared findings in mammary tumors with the findings in adjacent normal tissue [10-12,14], we cannot completely exclude genetic or endocrine alterations in our macro- and microscopically unremarkable samples.

Due to the retrospective nature of our study, we were not able to evaluate serum PRL levels of the patients. While previous reports indicated no difference in serum PRL level between bitches affected by benign or malignant mammary tumors [32,52], a recent study reported significantly higher serum PRL levels in bitches with malignant tumors [36]. A prospective study already in progress will show if female dogs affected by malignant tumors in fact have increased PRL levels in addition to decreased tumorous PRLR expression. However, as PRL serum levels are extremely difficult to compare in small study populations (pulsatile PRL secretion, massive fluctuation due to season, stage of estrous cycle and individual influences) and are moreover breed-dependent [2,53-59], a high patient number is needed to address this question.

## Conclusions

In summary, results of our preliminary study show that PRLR are expressed in canine mammary tumors, and expression seems to decrease with increasing malignancy. This finding is new and implicates different hypotheses. Firstly, decreasing PRLR expression might reflect dedifferentiation of malignant mammary epithelial cells. Secondly, it is possible that PRL influences canine mammary tumorigenesis despite decreasing receptor expression, via alterations in signaling pathways. The importance of PRL for canine mammary tumor development remains to be elucidated, and future studies are of interest.

## Competing interests

The authors declare that they have no competing interests.

## Authors’ contributions

EM was responsible of the study design, participated in data collection and interpretation of data and wrote the manuscript. SKF was responsible of data collection, participated in interpretation of data and drafting of the manuscript. MPK was responsible for design of laboratory work, performed the statistical analysis, and participated in data collection, interpretation of data and critical revision of the manuscript. CRB participated in study design, interpretation of data and critical revision of the manuscript. AB participated in interpretation of data and critical revision of the manuscript. FG did preliminary studies to establish PRLR immunohistochemistry and participated in interpretation of data and critical revision of the manuscript. IMR was responsible for overall supervision, participated in study design, interpretation of data and critical revision of the manuscript. All authors read and approved the final manuscript.
